# Leaf‐Like Graphene‐Oxide‐Wrapped Sulfur for High‐Performance Lithium–Sulfur Battery

**DOI:** 10.1002/advs.201500071

**Published:** 2015-06-10

**Authors:** Shouyi Yuan, Ziyang Guo, Lina Wang, Shuang Hu, Yonggang Wang, Yongyao Xia

**Affiliations:** ^1^ Department of Chemistry Shanghai Key Laboratory of Molecular Catalysis and Innovative MaterialsInstitute of New EnergyiChEM (Collaborative Innovation Center of Chemistry for Energy Materials)Fudan UniversityShanghai200433P.R. China

**Keywords:** carbon nanotube midribs, leaf‐like graphene oxides, lithium sulfur batteries, sulfur cathodes

## Abstract

Carbon/sulfur composites are attracting extensive attention because of their improved performances for Li–S batteries. However, the achievements are generally based on the low S‐content in the composites and the low S‐loading on the electrode. Herein, a leaf‐like graphene oxide (GO), which includes an inherent carbon nanotube midrib in the GO plane, is synthesized for preparing GO/S composites. Owing to the inherent high conductivity of carbon nanotube midribs and the abundant surface groups of GO for S‐immobilization, the composite with an S‐content of 60 wt% exhibits ultralong cycling stability over 1000 times with a low capacity decay of 0.033% per cycle and a high rate up to 4*C*. When the S‐content is increased to 75 wt%, the composite still shows a perfect cycling performance over 1000 cycles. Even with the high S‐loading of 2.7 mg cm^−2^ on the electrode and the high S‐content of 85 wt%, it still shows a promising cycling performance over 600 cycles.

## 1 Introduction

Lithium sulfur (Li–S) battery has recently attracted worldwide attentions due to its ultrahigh specific capacity of 1675 mAh g^−1^ based on sulfur, which far exceeds that of Li‐ion battery.^1^, ^2^, ^3^, ^4^, ^5^, ^6^, ^7^ In addition, sulfur is abundant in nature, low cost, and low toxicity.^1^, ^2^, ^3^, ^4^, ^5^, ^6^, ^7^ However, the insulating nature of sulfur (S) and its reaction products (i.e., Li_2_S), the large volume expansion from S to Li_2_S, along with the dissolution of lithium polysulfide intermediates (i.e., Li_2_S*_x_*, 4 ≤ *x* ≤ 8) into liquid electrolyte and the consequent shuttling effect between the anode and cathode, makes it generally display poor rate ability, limited cycle life and severe self‐discharge.^1^, ^2^, ^3^, ^4^, ^5^, ^6^, ^7^ Therefore, a variety of strategies have been pursued to circumvent the sulfur cathode problems, including optimization of organic electrolytes^8^, ^9^ and fabrication of sulfur‐conductive polymer composites^10^, ^11^ and sulfur–carbon‐based composites.^12^, ^13^, ^14^, ^15^, ^16^, ^17^, ^18^, ^19^, ^20^, ^21^, ^22^, ^23^, ^24^, ^25^, ^26^, ^27^, ^28^, ^29^, ^30^, ^31^, ^32^, ^33^, ^34^, ^35^, ^36^, ^37^, ^38^, ^39^, ^40^, ^41^, ^42^, ^43^, ^44^, ^45^ Among these approaches, porous‐carbon/sulfur composites^12^, ^13^, ^14^, ^15^, ^16^, ^17^, ^18^, ^19^, ^20^, ^21^, ^22^, ^23^ are more attractive because porous carbon can improve the electronic conductivity, accommodate the volume change, and suppress the dissolution of polysulfides. As a result, significant improvements in the utilization of sulfur and cyclability have been achieved by smartly designing porous‐carbon/sulfur composites.^12^, ^13^, ^14^, ^15^, ^16^, ^17^, ^18^, ^19^, ^20^, ^21^, ^22^, ^23^ However, limited sulfur content (usually below 70%)^12^, ^13^, ^14^, ^15^, ^16^, ^17^, ^18^, ^19^, ^20^, ^21^, ^22^, ^23^ in the carbon/sulfur (C/S) composites still limits the practical application of Li–S battery. Especially, a lot of promising performances were achieved by low mass loading of sulfur on the electrode.^13^, ^16^, ^20^, ^21^, ^23^ As highlighted in the recent review on Li–S batteries,^24^ the cathode is required to have a sulfur content of 70% by weight and reasonable sulfur loading equaling to a specific areal capacity of 2–3 mAh cm^−2^. In addition, hydrophilic lithium polysulfide intermediates can diffuse out of hydrophobic porous carbon during long‐term cycling, which still results in capacity fading.^46^

Very recently, many efforts have been made to develop graphene oxide/sulfur (GO/S) composites cathode for Li–S battery,^25^, ^26^, ^27^, ^28^, ^29^, ^30^, ^31^, ^32^, ^33^, ^34^, ^35^ because GO consists of a basal plane decorated mostly with epoxide and hydroxyl groups, in addition to carbonyl and carboxyl groups, which are located on the edges. Zhang and co‐workers successfully demonstrated that these functional groups on the surface of GO play an important role for immobilizing the sulfur and its discharge products.^25^ According to their report, strong chemical interaction between S and the functional groups on GO happens and S can partially reduce the GO. Especially, according to very recent investigations,^41^, ^46^ the hydrophilic surface groups of GO should be quite important for immobilizing the hydrophilic lithium polysulfide intermediates. Furthermore, GO network can also accommodates the volume change of the electrode during the Li–S electrochemical reaction. Then, more and more researches have focused on GO/S composites and modified GO/S composites, and further improvements in cyclability of Li–S battery have been achieved by smartly designing GO/S composites in recent years.^25^, ^26^, ^27^, ^28^, ^29^, ^30^, ^31^, ^32^, ^33^, ^34^, ^35^ However, one of the most salient problems of GO also arises from the functional groups on the surface that lead to poor electronic conductivity.^46^ Therefore, the achieved rate performances in most previous reports about GO/S composites are generally below the rate of 2*C* (1*C* = 1675 mA g^−1^).^25^, ^26^, ^27^, ^28^, ^29^, ^30^, ^31^, ^32^, ^33^, ^34^, ^35^ On the contrary, reduced GO (RGO) or graphene (G) with higher electronic conductivity was also employed to prepare RGO/S or G/S composites for improving rate performance.^36^, ^37^, ^38^, ^39^, ^40^, ^41^, ^42^, ^43^, ^44^ However, RGO or G does not have the abundant functional groups that can bind sulfur and its discharge products. In addition, similar with previous reports about porous‐carbon/S composites,^12^, ^13^, ^14^, ^15^, ^16^, ^17^, ^18^, ^19^, ^20^, ^21^, ^22^, ^23^ the sulfur content in the GO/S and RGO/S (or G/S) composites and sulfur loading on the electrode is still low.^25^, ^26^, ^27^, ^28^, ^29^, ^30^, ^31^, ^32^, ^33^, ^34^, ^35^, ^36^, ^37^, ^38^, ^39^, ^40^, ^41^, ^42^, ^43^, ^44^ In addition, some efforts^34^ have been made to mix high conductivity carbon nanotube (CNT) with GO to improve the conductivity of GO. In theory, the mixture of CNT/GO can unite the advantages of CNT and GO. However, in practice, the CNT cannot be intimately contacted with the layered GO. As a result, the improvement of battery is still limited.^34^ Therefore, it is highly desired to develop perfect GO with both various functional groups and improved electronic conductivity for the design of advanced GO/S composites. It is also highly desired to increase the sulfur content in the composites and to enhance the sulfur loading on the electrode for practical application.

In present work, we designed and synthesized a new kind of leaf‐like GO, which includes an inherent CNT midrib and layered GO grown around the inherent CNT‐midrib, from vapor grown carbon fiber (VGCF) by a conventional Hummer's method. Then, it was employed to prepare the GO/S composites that were then investigated as the cathode composites for Li–S battery for the first time. In our composites, a CNT midrib inherently grows in the basal plane of GO, which can effectively unite the advantages from CNT and GO. Owing to the improved electronic conductivity from the CNT‐midrib and the abundant functional groups for sulfur immobilization, the as‐prepared GO/S composites display better performance (i.e., cycling performance and rate performance) than previous reports. Especially, with the increase of S wt% in the composite and the mass loading of S on the electrode, the corresponding Li–S batteries still displays promising performance.

## 2 Results and Discussion

### 2.1 Characterization of Leaf‐Like GO and Leaf‐Like GO/S Composites

The leaf‐like GO was experimentally realized from VGCF by a conventional Hummer's method (see the Experimental Section). The structure of VGCF can be summarized as: multilayered G surrounds and wraps an axis of CNT to form a graphite rod, which is confirmed by both scanning electron microscopy (SEM) (Figure S1a–c, Supporting Information) and transmission electron microscopy (TEM) images (Figure S2a–e, Supporting Information). By oxidation via a conventional Hummer's method, the graphite rod (i.e., VGCF) is expanded and exfoliated along the CNT axis to become leaf‐like GO. The CNT remains in the basal plane of GO to form a natural midrib for GO leaf, which can offer a natural electron pathway for GO leaf. The detail preparation process of leaf‐like GO has been illustrated in our previous report.^47^ According to the preparation process, the CNT midribs is embedded in the basal plane of GO sheet. The leaf‐like GO/S composites were then synthesized via a solution‐based method followed by heat treatment in argon at 155 °C. (Detail experimental procedures are given in the Experimental Section).

**Figure 1:**
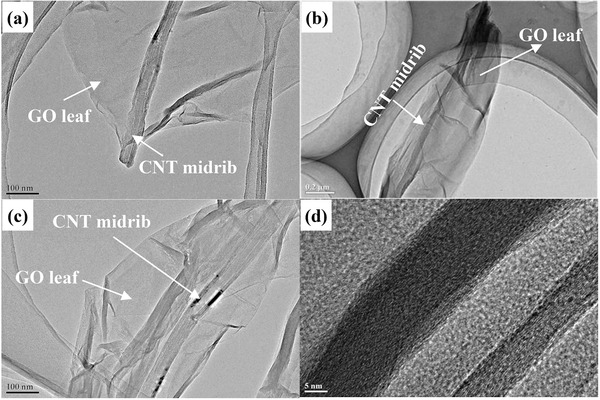
TEM images of leaf‐like GO at different magnifications.

**Figure** **1** displays the TEM images of leaf‐like GO. A CNT midrib can be clearly found in the basal plane of GO. As further confirmed by high resolution‐transmission electron microscopy (HR‐TEM) images (Figure 1d), the midrib of the leaf‐like GO is a typical CNT with an outer diameter of 30–40 nm and an inner diameter of 10 nm. The outer diameter of the CNT is much smaller than that of pristine VGCF (Figure S1c, Supporting Information), indicating that the spiral structure of VGCF has been peeled off along with the CNT axis. This point is also confirmed by the SEM images of leaf‐like GO (Figure S3, Supporting Information). In addition, it should be also noted in Figure 1d that the wall thickness on the one side is much greater than the other side. The thicker area is due to the remaining partial connection of the exfoliated layers with the central tube. All the TEM images (Figure 1a–c) verify the structure of leaf‐like GO composites that can be summarized as: GO layer with an inherent CNT midrib. The electronic conductivity of leaf‐like GO powder is 0.7 S cm^−1^, which is close to the conductivity of CNT powder (35 S cm^−1^) and much higher than that of conventional GO (8 × 10^−5^ S cm^−1^) powder prepared by Hummer's method.^47^

**Figure 2:**
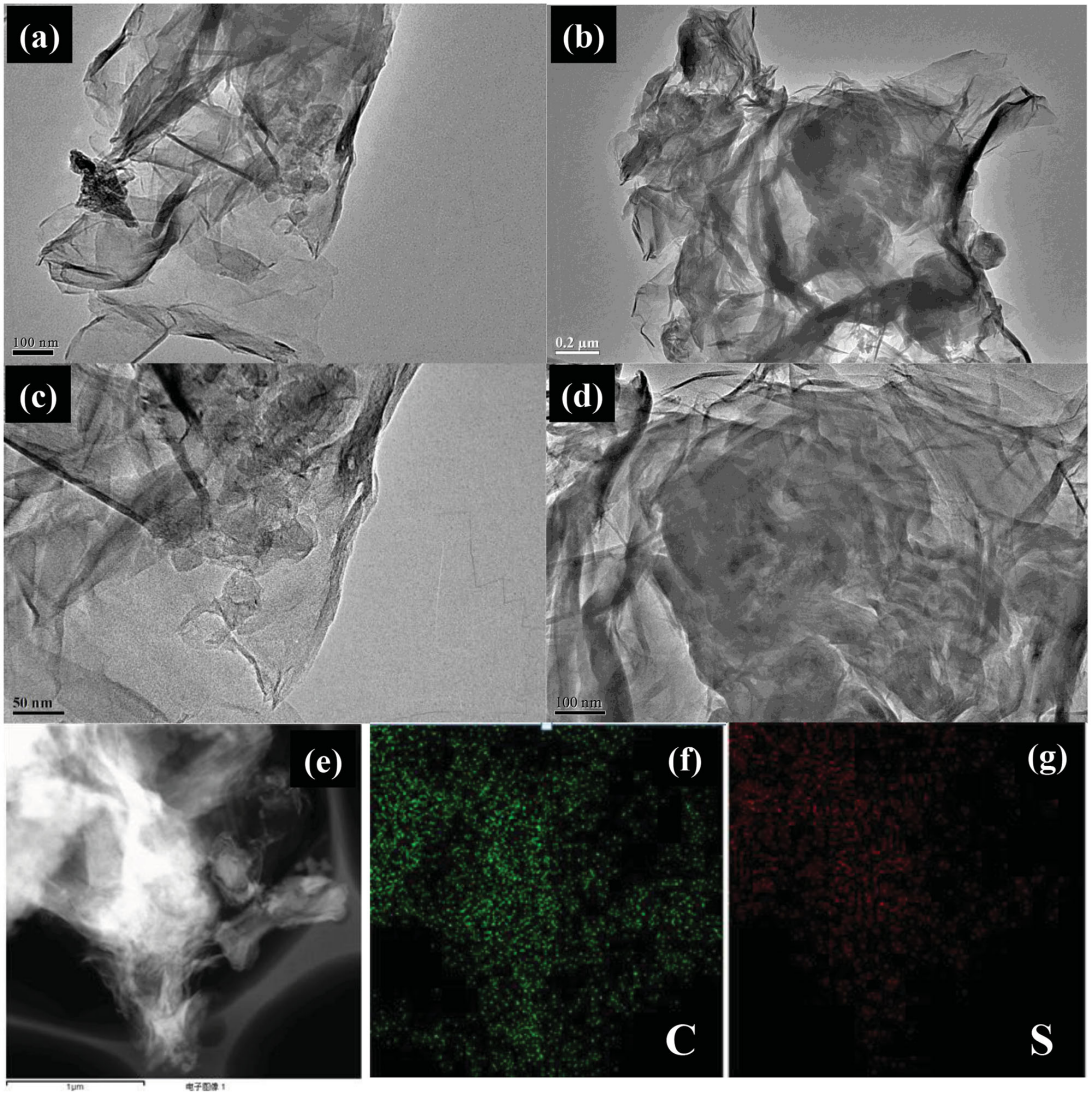
TEM images and STEM mapping equipped in TEM of leaf‐like GO/S. a–d) TEM images of leaf‐like GO/S composites. e) STEM images of leaf‐like GO/S. f) STEM mapping of C. g) STEM mapping of S.

**Figure 3:**
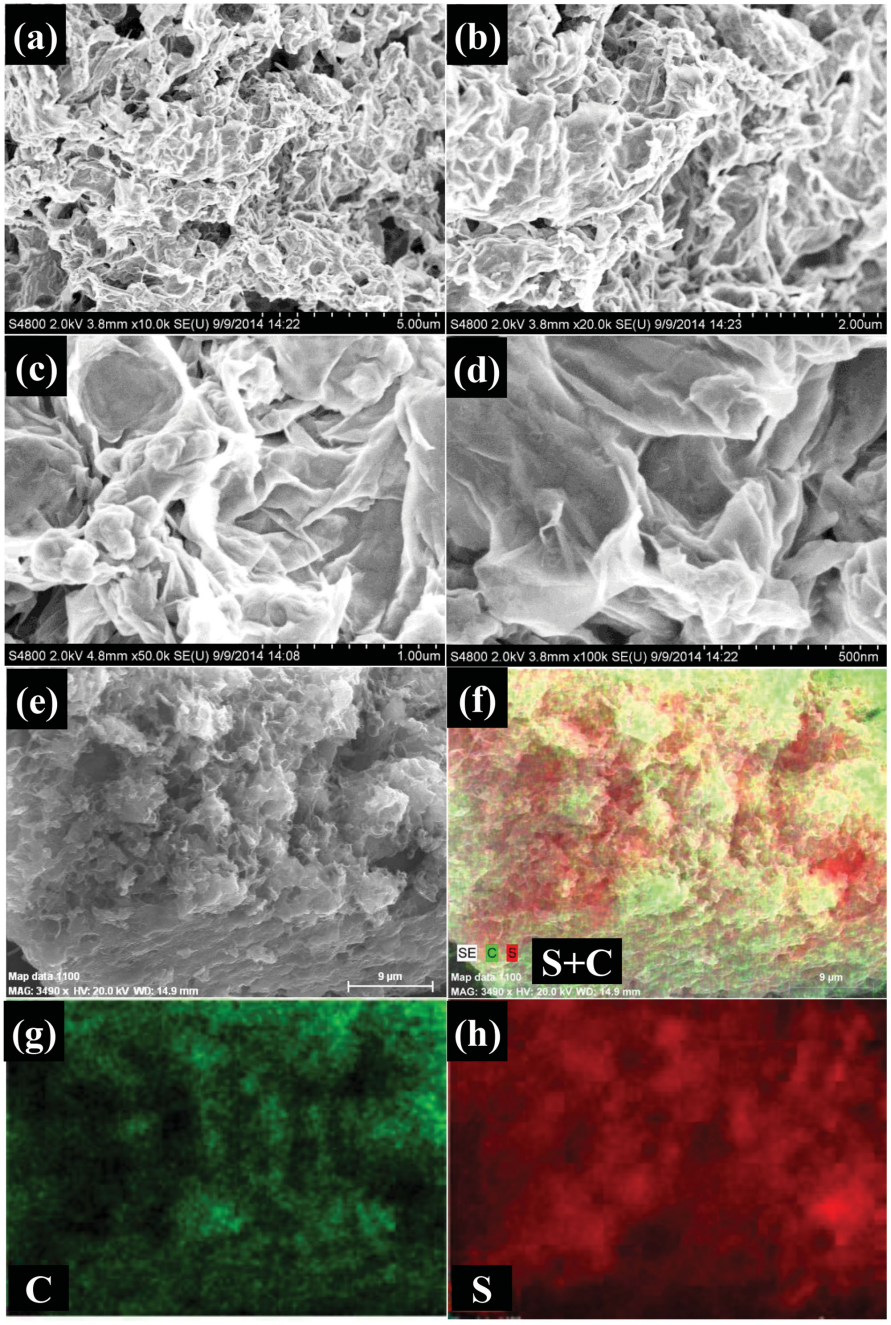
SEM images and EDX mapping equipped in SEM of leaf‐like GO. a–d) SEM images of leaf‐like GO/S composites. e) EDX images of leaf‐like GO/S. f) EDX mapping of C and S. g) EDX mapping of C. h) EDX mapping of S.

**Figure** **2**a–d shows the TEM images of leaf‐like GO‐wrapped sulfur particles. SEM images with different magnitudes are also given in **Figure** **3**a–d. In both SEM and TEM images, no obvious bulk sulfur is found, which suggests the uniformed distribution of sulfur in the GO leaf. In addition, scanning transmission electron microscopic (STEM) mapping‐equipped TEM (Figure 2e–g) and the element mapping by energy‐dispersive X‐ray (EDX) analysis equipped in SEM (Figure 3e–h) further verify that sulfur particles are coated by the leaf‐like GO layer. Notably, even after strong ultrasonication to disperse samples in ethanol for TEM test, the sulfur particles were still anchored on the surface of GO, indicating good adhesion of sulfur particles on the surface of leaf‐like GO.

**Figure 4:**
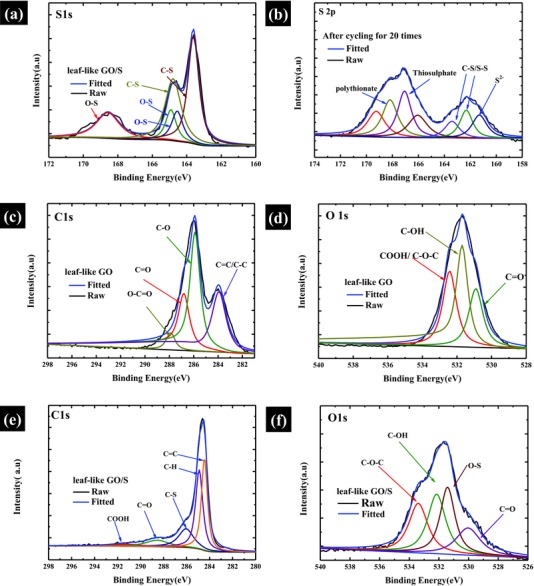
XPS spectrum of leaf‐like GO and leaf‐like GO/S. a) S 2*p* XPS spectrum of leaf‐like GO/S before cycling. b) S 2*p* XPS spectrum of leaf‐like GO/S after cycling for 20 times. c) C 1*s* XPS spectrum of leaf‐like GO. d) O 1*s* XPS spectrum of leaf‐like GO. e) C 1*s* XPS spectrum of leaf‐like GO/S. f) O 1*s* XPS spectrum of leaf‐like GO/S.

In order to demonstrate the strong binding ability between leaf‐like GO and S and the partial reduction of leaf‐like GO in the leaf‐like GO/S composites, X‐ray photoelectron spectroscopy (XPS) was also carried out on the leaf‐like GO and leaf‐like GO/S composites. **Figure** **4**a–f shows the XPS spectrum of leaf‐like GO and leaf‐like GO/S composites. Figure 4a,b exhibits the S 2*p* XPS spectrum for leaf‐like GO/S before cycling (Figure 4a) and leaf‐like GO/S after cycling for 20 times (Figure 4b). As shown in Figure 4a, a strong peak at ≈163.6 eV is observed, which can be assigned to active S species with the C—S and S—S bond, while the shoulder peak at ≈164.8 eV can be fitted by three peaks, which is assigned to C—S bond and O—S bond respectively.^48^ Furthermore, the peak at ≈168.4 eV can be assigned to the O—S bond arising from the sulphate species formed by oxidation of S in air.^48^ It can be identified the presence of C—S and O—S bonds in the S 2*p* XPS spectrums. These chemical bonds between leaf‐like GO and S can largely trap the S on the surface of GO and limit diffusion of lithium polysulfide into the electrolyte.^48^ Figure 4b exhibits the S 2*p* XPS spectrums after cycling for 20 times. Notably, the peak intensity from 171.1 to 165.0 eV assigned to polythionate (≈168.2 eV) and thiosulphate(≈167.2 eV), respectively becomes much stronger, while the peaks corresponding to active S species with C—S bond and O—S bond from 164.0 to 160.0 eV have weakened. The result indicates that some of the active S species has been converted into the polythionate and thiosulphate during cycling.^48^ The as‐formed polythionate and thiosulphate species during cycling can serve as highly efficient polysulfide mediator to limit the diffusion of lithium polysulfides, which is confirmed by recent report.^49^

To confirm the reduction of GO, XPS spectrums of C 1*s* and O 1*s* of leaf‐like GO and leaf‐like GO/S are also carried out. (Figure 4c–f) It can be identified in the C 1*s* spectrum (Figure 4c,e) that the intensity peak corresponding to sp^2^ hybrid carbon (≈284.1 eV) increases from leaf‐like GO to leaf‐like GO/S, while some of the peaks arising from the C—O species(i.e., 287.2 eV for C=O, 286.1 eV for C—O, and 288.6 eV for O—C=O) in the leaf‐like GO/S exhibit opposite trend. The results suggest that some of the O functional groups in the leaf‐like GO/S composites has been removed in the leaf‐like GO/S composites, indicating that the leaf‐like GO has been partially reduced in the leaf‐like GO/S composites.^41^ To further confirm the reduction of leaf‐like GO, we have also calculated the atomic ratio of C/O, since the reduction degree of GO is judged by the atomic ratio of C/O.^50^ The ratio is calculated based on the peak areas of C 1*s* and O 1*s* spectra and the sensitivity factors (0.25 for C and 0.66 for O).^50^ The ratio of C/O is 1.69 for leaf‐like GO and 3.42 for leaf‐like GO/S composites. All the evidences shown here clearly indicate the partial reduction of leaf‐like GO in the leaf‐like GO/S composites. To further demonstrate the structure of leaf‐like GO and leaf‐like GO/S composites, Fourier transform infrared spectroscopy, the Raman spectroscopy, X‐ray diffraction, and Brunauer−Emmett−Teller measurement were carried out (see Figure S4 and corresponding discussion in the Supporting Information).

### 2.2 Electrochemical Performance of Li–S Batteries with a Sulfur Content of 60 wt% in Composites

In order to clarify the structure benefit of leaf‐like GO/S composites for Li–S battery, a series of electrochemical measurements were carried out. The leaf‐like GO/S composites with a sulfur content of 60 wt% (see TG analysis in Figure S5 in the Supporting Information) were tested to evaluate the effect of leaf‐like GO/S on the electrochemical performance. The electrochemical performance of the leaf‐like GO/S composites as the cathode of Li–S battery were evaluated with CR2016 coin cell with average total mass loading of 2 mg cm^−2^ ((80 wt% GO/S + 10 wt% super P + 10 wt% polyvinylidene fluoride binder (PVdF)) on the electrode. The average S‐loading is about 1 mg cm^−2^ (2 mg cm^−2^ × 80% × 60%). Detail procedures for fabrication of electrodes and batteries are given in the Experimental Section. The battery was cycled within a voltage ranging from 1.70 to 2.60 V. Specially, the voltage of cells tested at current rate of 2*C* and 4*C* ranges from 1.40 to 3.00 V given that the polarization is much larger at higher current density.

**Figure 5:**
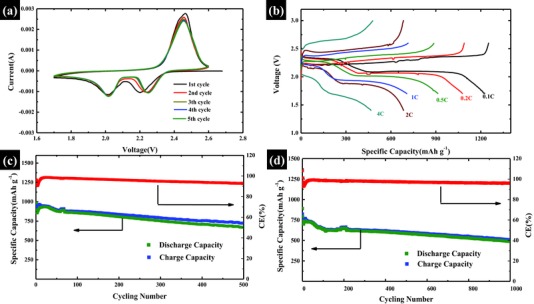
Electrochemical performance of leaf‐like GO/S composites with sulfur content of 60 wt%. a) Cyclic voltammetry plots at a scan rate of 0.1 mV s^−1^ with 1.7–2.6 V voltage windows. b) Galvanostatic charge/discharge profiles of the battery with different rates ranging from 0.1*C* to 4*C* (1*C* = 1670 mAh g^−1^). c) Cycling performance at the current rate of 0.5*C*. d) Cycling performance at the current rate of 1*C*.

**Figure** **5**a displays cyclic voltammograms (CV) of the leaf‐like GO/S composites cathode for the initial five cycles between 1.70 and 2.60 V at a scan rate of 0.10 mV s^−1^. Two conventional reduction peaks at 2.05 and 2.28 V, which can be assigned to the multistep reduction mechanism of element S, are observed. The reduction peak at 2.28 V is the reduction process from elemental S to long chain polysulfides, while the reduction peak at 2.05 V can be assigned to the reduction process from long chain lithium polysulfides to short chain lithium sulfides. The overlapping oxidation peak at 2.47 V refers to the reverse process. Notably, the cathodic peaks in initial CV curve do not finely coincide with the following several circles. That is likely due to the activation of the battery and the gradual enhancement of the sulfur utilization on the first cycle. From the second cycle, both the CV peak positions and areas remain almost unchanged, suggesting relatively good chemical stability and capacity retention. Figure 5b shows the Galvanostatic charge/discharge profiles of the battery with different rates ranging from 0.1*C* to 4*C* (1*C* = 1670 mAh g^−1^). The discharge/charge profile shows two discharge plateaus at 2.35 and 2.08 V, corresponding to the two reduction peaks in CV curve. The battery delivers a reversible discharge capacity of 1250 mAh g^−1^ at current rate of 0.1*C* that slowly reduced to 1130 mAh g^−1^ at 0.2*C*, 930 mAh g^−1^ at 0.5*C*, 753 mAh g^−1^ at 1*C*, and 685 mAh g^−1^ at 2*C*. Even at high rate of 4*C* (6680 mA g^−1^), the leaf‐like GO/S cathode still can deliver a capacity of 468 mAh g^−1^, which is almost the best rate performance of C/S composite cathode.^8^, ^10^, ^11^, ^12^, ^14^, ^15^, ^16^, ^17^, ^18^, ^19^, ^20^, ^21^, ^22^, ^25^, ^26^, ^27^, ^28^, ^29^, ^30^, ^31^, ^32^, ^33^, ^34^, ^45^, ^46^, ^48^, ^51^, ^52^, ^53^

For the practical application of Li–S battery, excellent capacity retention over long cycles is indispensable. Figure 5c, 5d gives the cycling performance of batteries with leaf‐like GO/S composites cathode at current rates of 0.5*C* and 1*C*. Figure 5c shows the cycling performance of 0.5*C*, an initial discharge capacity of 984 mAh g^−1^ is achieved. After three cycles, the discharge capacity stabilizes at 854 mAh g^−1^. Then, the capacity decreased to 830 mAh g^−1^ after 100 cycles, representing a capacity loss of 2.8%. After 300 cycles, the capacity remained 753 mAh g^−1^, indicating a capacity loss of 11.8%. Even after 500 cycles, the battery still remained a capacity of 670 mAh g^−1^. The capacity degradation is only 0.043% per cycle. When the current rate increases to 1*C* (Figure 5d), the leaf‐like GO/S composites exhibited a stable cycling performance over 1000 cycles. The battery displayed an initial discharge capacity of 892 mAh g^−1^. After cycling for three times, the capacity stabilized at 736 mAh g^−1^. A capacity of 613 and 583 mAh g^−1^ can be obtained after 300 and 500 cycles, corresponding to a capacity retention of 83.9% and 79.6% of the stabilized capacity. Even after cycling for 1000 times, a capacity of 490 mAh g^−1^ is still achieved. The capacity retention of the leaf‐like GO/S composites over 1000 cycles is 67%, which corresponds to a very small capacity decay of 0.033% per cycle. In addition, the columbic efficiency of over 1000 cycles maintained over 95%, which demonstrates the excellent ability to suppress the shuttling effect of polysulfides. The cycling performance of the battery at 0.2*C* and 2*C* is also given in the Supporting Information, which is also displayed a superior cycling performance (Figure S6, Supporting Information). All the capacity retention and capacity degradation are calculated from the third cycle after achieving stabilized capacity. The achieved cycling ability in Figure 5c,d is much better than that of most previous reports.^8^, ^9^, ^10^, ^11^, ^12^, ^13^, ^14^, ^15^, ^16^, ^17^, ^18^, ^19^, ^20^, ^21^, ^22^, ^23^, ^24^, ^25^, ^26^, ^27^, ^28^, ^29^, ^30^, ^31^, ^32^, ^33^, ^34^, ^35^, ^36^, ^37^, ^38^, ^39^, ^40^, ^41^, ^42^, ^48^ Only very recently, Cui and co‐workers^51^ successfully demonstrated that the TiO_2_/S yolk–shell composites can be cycled over 1000 times with a capacity decay of 0.033% per cycle. However, the sulfur loading on their electrode is only 0.4–0.6 mg cm^−2^, which is much lower than that of ours. In addition, we also investigate the self‐discharge of the batteries with a sulfur content of 60 wt% (see Figures S7 and S8 in the Supporting Information). Based on the result of self‐discharge investigation, leaf‐like GO/S composites can largely limit the self‐discharge of Li–S battery. However, to further reduce the self‐discharge of Li–S batteries, a synergy of methods are still needed such as optimizing the electrolyte,^7^, ^8^ designing new battery structure,^52^, ^53^ protecting Li anode,^54^, ^55^ and spatially controlling sulphur species deposition.^56^ For comparison, conventional GO/S composite was synthesized and investigated as electrode for Li–S battery (Figure S9, Supporting Information). It can be detected that the specific capacity of conventional GO/S composites at the same rate of 1*C* is much lower than that of leaf‐like GO/S composites, which is mainly due to the poor conductivity of conventional GO.

Above all, the achieved long‐term operation up to 1000 cycles with extremely low degradation rate of 0.033% per cycle and high rate performance up to 4*C* can clearly demonstrate the superior performance of leaf‐like GO/S composites cathode. The improved cycling performance of leaf‐like GO/S composites should be attributed to synergic effects including excellent ability of GO to immobilize polysulfides and rich wrinkles to accommodate the volume expansion, while the excellent rate performance should be ascribed to the inherent CNT midrib on the GO leaf to enhance the conductivity.

### 2.3 Electrochemical Performance of Li–S Batteries with Higher Sulfur Content

To further demonstrate the excellent performance of leaf‐like GO/S composites, we also performed electrochemical measurement with higher sulfur content. A series of leaf‐like GO/S composites with higher sulfur content were synthesized by adjusting the mass of sulfur and leaf‐like GO. The sulfur content in the leaf‐like GO/S composites is 75 and 85 wt% respectively (see TG analysis in Figure S5 in the Supporting Information). The electrochemical performance of the leaf‐like GO/S composites with the sulfur content of 75 and 85 wt% were also evaluated with CR2016 coin cell with an average total mass loading of 2 mg cm^−2^ (80 wt% GO/S + 10 wt% super P + 10 wt% PVdF) on the electrode. The S‐loading is about 1.2 mg cm^−2^ (2 mg cm^−2^ × 80% × 75%) and about 1.4 mg cm^−2^ (2 mg cm^−2^ × 80% × 85%), respectively. The voltage profiles of leaf‐like GO/S composites with different sulfur contents (i.e., 60, 75, and 85 wt%) at different rates are given in Figure S10 (Supporting Information) for comparison.

**Figure 6:**
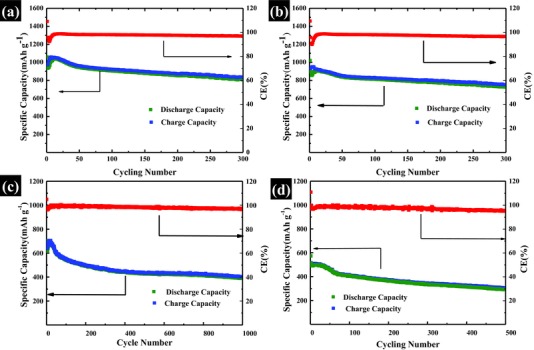
Cycling performance with higher sulfur content. a) Cycling performance at 0.2*C* with a sulfur content of 75 wt%. b) Cycling performance at 0.2*C* with a sulfur content of 85 wt%. c) Cycling performance at 1*C* with a sulfur content of 75 wt%. d) Cycling performance at 1*C* with a sulfur content of 85 wt%.

**Figure** **6**a–d displays the cycling performance of the composites with a sulfur content of 75 and 85 wt% at current rates of 0.2*C* (Figure 6a,b) and 1*C* (Figure 6c,d). The leaf‐like composites with sulfur content of 75 and 85 wt% show almost similar specific capacity and stable cycling performance over 300 cycles at the current rate of 0.2*C*. Furthermore, when the sulfur content increases to 75 and 85 wt%, the achieved capacity at 0.2*C* of leaf‐like GO/S composite can still reach 950 and 890 mAh g^−1^ (see Figure 6a,b and Figure S10 in the Supporting Information), which is close to that of leaf‐like GO/S composites with a sulfur content of 60 wt% (see Figure S10 in the Supporting Information). When the current density increase to 1*C*, the leaf‐like GO/S composites with 75 wt% sulfur loading still exhibited excellent cycling performance over 1000 cycles at the current density of 1*C*. The battery delivered an initial discharge capacity of 701 and stabilized at 615 mAh g^−1^ after cycling for 3 times. The battery held a capacity of 425 mAh g^−1^ after cycling for 500 times and even after 1000 cycles, the battery still displayed a capacity 390 mAh g^−1^, representing the superior ability for absorbing polysulfides and adhering to Li_2_S. The capacity retention over 1000 cycles is 62% with a low decay rate of 0.038% per cycle. Moreover, the columbic efficiency over 1000 cycles is closed to 100%, indicating the excellent ability to suppress the shuttling effect. Figure 6d gives the cycling performance with sulfur loading of 85 wt% in the leaf‐like GO/S composites. As shown in Figure 6d, even with an ultrahigh sulfur content of 85 wt% in the leaf‐like GO/S composites, the battery still display superior cycling performance over 500 cycles. The battery gives an initial discharge of 573 mAh g^−1^, which rapidly stabilized at 493 mAh g^−1^ after 3 cycles. From 4th cycle to 500th cycle, the capacity slightly drops from 493 to 290 mAh g^−1^. The capacity retention of the battery over 500 cycles is 59.2% with a capacity degradation rate of 0.081% per cycle. It should be noted that few previous reports can achieve superior performance at a high sulfur content of 85 wt%. This point can further demonstrate the high performance of our leaf‐like GO/S composite. The cycling performance with a sulfur content of 75 and 85 wt% at current rate of 0.5*C* is also given in the Supporting Information (Figure S11, Supporting Information). To our best knowledge, the highest sulfur content in C/S composites is 82 wt%.^31^ However, the capacity decay rate of the composites with a sulfur content of 82 wt% is about 0.28% per cycle,^31^ which is much higher than ours.

### 2.4 Electrochemistry Performance of Leaf‐Like GO/S Composites with Different Mass Loading

**Figure 7:**
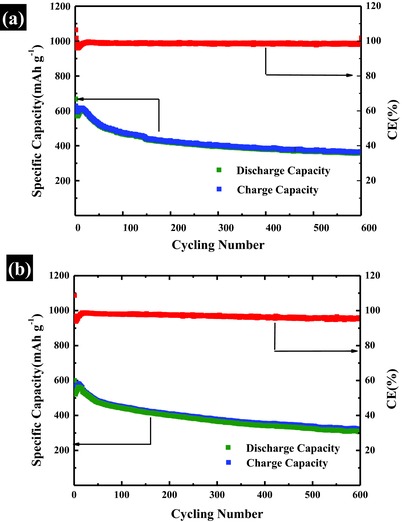
Cycling performance with different total mass loading. a) Cycling performance with a total mass loading of 2 mg cm^−2^ on the electrode. b) Cycling performance with a total mass loading of 4 mg cm^−2^ on the electrode.

Finally, we discuss the effect of total mass loading of the electrode on the performance of the battery. In this section, leaf‐like GO/S composites with a sulfur content of 85 wt% were employed to investigate the effect of total mass loading of electrode on the performance of batteries. Electrodes with total mass (80 wt% GO/S + 10 wt% Super P + 10 wt% PVdF) loading of 2 mg cm^−2^ (S‐loading = 1.4 mg cm^−2^) and 4 mg cm^−2^ (S‐loading = 2.7 mg cm^−2^) were cycled at current rate of 0.5*C* for comparison. Figure S12 (Supporting Information) displays voltage profile with different total loadings both at 10th cycle. **Figure** **7** shows the cycling performance of leaf‐like GO/S composites with total mass loading of 2 mg cm^−2^ (S‐loading = 1.4 mg cm^−2^, Figure 7a) and 4 mg cm^−2^ (S‐loading = 2.7 mg cm^−2^, Figure 7b) on the electrode at current rate of 0.5*C*.

As shown in Figure 7b, the battery can still run over 600 cycles even at high total mass loading of 4 mg cm^−2^ (S‐loading = 2.7 mg cm^−2^). It should be noted that high mass loading of S is very important for the practical application of Li–S battery. It is because that although the specific weight capacity (mAh g^−1^) of S is much higher than that of conventional cathode materials for Li‐ion battery, the low gravity density of S and large amount of inactive carbon supporter largely limit the volumetric capacity and the areal capacity of C/S composite electrode in practical application. As highlighted in Zhang's review article on Li–S batteries,^24^ to retain the advantage of sulfur's high energy density, the cathode is required to have a sulfur content of at least 70 wt% by weight and reasonable sulfur loading equaling to a specific areal capacity of 2–3 mAh cm^−2^. As mentioned above, the mass loading of S in this experiment is 2.7 mg cm^−2^. Based on the initial discharge capacity of 600 mAh g^−1^ achieved at the rate of 0.5*C* (Figure 7), the calculated special areal capacity of leaf‐like GO/S composite electrode is around 3.0 mAh cm^−2^ that can also meet the requirement. Even after cycling for 600 cycles, the battery still gives a specific areal capacity of 1.6 mAh cm^−2^. However, the specific areal capacity in most previous reports^13^, ^16^, ^20^, ^21^, ^23^, ^26^, ^28^, ^30^, ^39^, ^40^, ^43^, ^45^, ^51^ is lower than 2 mAh g^−1^ due to the low sulfur loading in the electrodes (see Table S1 in the Supporting Information and corresponding discussion). Moreover, some previous superior cycling performances over 500 cycles^20^, ^43^, ^45^, ^51^ are achieved by low sulfur loading, which is usually lower than 1 mg cm^−2^ (Table S1, Supporting Information), while in our present work, the electrode with 2.7 mg cm^−2^ sulfur loading can still run over 600 cycles. In addition, the leaf‐like GO/S composite with a sulfur content of 75 wt%, which is corresponding to sulfur loading of 1.2 mg cm^−2^ (sulfur loading = 2 × 75% × 80% = 1.2 mg cm^−2^) in the electrode, can still run over 1000 cycles with an extremely low capacity decay of 0.038% per cycle (see Figure 6).

## 3 Conclusion

In summary, three important factors contribute to the excellent performance of leaf‐like GO/S composites. First, various functional groups on the leaf‐like GO can largely immobilize the sulfur and its discharge products. Moreover, an inherent CNT midrib in the basal plane of GO can provide a natural electron pathway that largely improve the conductivity of GO. Finally, rich wrinkles of GO provide enough room for the volume expansion of sulfur. We wish that the results presented here would encourage further studies of designing cathode structure for Li–S batteries, although many challenges in realizing practical devices still remain.

## 4 Experimental Section

*Synthesis of Leaf‐Like GO Composites*: The leaf‐like GO were synthesized from VGCF by a conventional Hummer's method according to our previous report.^47^ Briefly, pristine VGCF (2 g) was added into concentrated H_2_SO_4_ at a temperature of 0 °C using ice bath. Initially, NaNO_3_ (1 g) and KMnO_4_ (6 g) were added slowly into the mixture with constant stirring and cooling so that the temperature of the mixture did not reach 20 °C. Subsequently, the ice bath was removed and the mixture was heated to 35 °C in water bath and maintained at this temperature for half an hour. Then, 92 mL deionized (DI) water was put into the mixture slowly so that the temperature of the mixture increased to 98 °C and maintained at this temperature for 30 min using oil bath, and then further diluted the mixture with DI water (280 mL) and added H_2_O_2_ (3 mL) into the mixture. Consequently, the precipitate was collected and washed several times with DI water using centrifuge. Eventually, the precipitate was redispersed into DI water to form a homogeneous solution. For comparison, conventional GO were also synthesized from graphite by a conventional Hummer's method.

*Synthesis of Leaf‐Like GO/S Composites*: The leaf‐like GO/S composites were synthesized via a solution‐based method followed by low‐temperature heat treatment. Briefly, nanosized sulfur particles were synthesized by adding HCl (10 m 8 mL) into a solution of Na_2_S_2_O_3_ (0.04 m 100 mL) with low concentration of polyvinylpyrrolidone (PVP, *M*_w_ = 24 000 0.02 wt%) under constant stirring at room temperature for 2 h. Then, the precipitate were collected, washed for several times by centrifuge, and then dried at 60 °C under vacuum overnight. For synthesis of the leaf‐like GO/S composites, the as‐prepared leaf‐like GO solution was sonicated for half an hour to form a homogeneous solution. The as‐prepared nanosized sulfur particles were also dispersed into DI water and sonicated for half an hour to form a uniformed solution. Then, the leaf‐like GO/S composites were synthesized by adding the nanosized sulfur particles dispersion into the as‐prepared leaf‐like GO solution and then stirred for 2 h. The sulfur content in the leaf‐like GO can be altered by adjusting the ratio of leaf‐like GO mass and sulfur mass. The precipitate was collected using centrifuge and then dried in the air under 60 °C for overnight. Finally, the product was heat‐treated at 155 °C for 12 h in a sealed vessel filled with argon to get a homogeneous black powder. For comparison, GO/S composite were also prepared by the same procedure.

*Characterization*: The surface morphologies of the above composites were characterized by SEM (FE‐SEM S‐4800) and TEM (JEOL JEM‐2100 F microscope (Japan) operated at 200 kV). Elemental mapping was performed using energy‐dispersive X‐ray spectroscopy (ED) equipped in SEM and STEM equipped in TEM. XPS measurements were carried out on a XSAM800 Ultra spectrometer.

*Electrochemical Measurements*: To fabricate the sulfur cathode, 80 wt% leaf‐like GO/S composites, 10% Super P and 10 wt% PVdF were initially mixed in a *N*‐methyl‐2‐pyrrolidine solution, and the resulting slurry was coated on an Al foil. If no specially mention, the total mass loading of electrode is approximately 2 mg cm^−2^ for each electrode. In order to compare the effect of different mass loading on the battery performance, cathodes with higher total mass loading of 4 mg cm^−2^ were also prepared. Finally, the electrode was dried for 12 h at 50 °C under vacuum to remove residual solvent. CR2016‐type coin cells were fabricated in an argon‐filled glove box using lithium foil as the counterpart electrode. These two electrodes were separated by a Celguard separator dipping with DOL/DME(1:1) –(1 m) LiTFSI electrolyte containing 1 wt% LiNO_3_ additives. The cells were cycled in a LAND cycler Wuhan Land Electronic Co. Ltd. Galvanostatic charge–discharge measurement within a voltage ranging from 1.7–2.6 V. The voltage of cells tested at current rate of 2*C* and 4*C* ranges from 1.4 to 3.0 V. Specially, electrode for ex situ XPS analysis were prepared by discharging at 0.5*C* in (1 m)‐LiClO_4_ (to avoid sulfur contributions from LiFTSI) in DOL/DME (1:1). Before the XPS test, the electrode was washed several times in pure DME solvent to move the electrolyte salts and dried for several days under vacuum at room temperature to remove the solvent. Cyclic voltammograms were conducted using CHI 660 Electrochemical Workstation.

## Supporting information

As a service to our authors and readers, this journal provides supporting information supplied by the authors. Such materials are peer reviewed and may be re‐organized for online delivery, but are not copy‐edited or typeset. Technical support issues arising from supporting information (other than missing files) should be addressed to the authors.

Supplementary
