# Supplementary material for: Leaf‐Like Graphene‐Oxide‐Wrapped Sulfur for High‐Performance Lithium–Sulfur Battery
Source: Adv Sci (Weinh). 2015 Jun 10;2(8):1500071. doi: 10.1002/advs.201500071 (PMC5115426; doi:10.1002/advs.201500071)
Supplement: Supplementary file 1 — Supplementary [file ADVS-2-0c-s001.pdf]

Copyright WILEY-VCH Verlag GmbH & Co. KGaA, 69469 Weinheim, Germany, 2015.

Supporting Information

for *Adv. Sci.*, DOI: 10.1002/advs. 201500071

Leaf-Like Graphene-Oxide-Wrapped Sulfur for High-Performance Lithium–Sulfur Battery

Shouyi Yuan, Ziyang Guo, Lina Wang, Shuang Hu, Yonggang Wang, and Yongyao Xia**

WILEY-VCH

WILEY-VCH Verlag GmbH & Co. KGaA, 69469 Weinheim, Germany, 2013.

Supporting Information

Leaf-Like Graphene Oxide Wrapped Sulfur for High Performance Lithium–Sulfur Battery

Shouyi Yuan, Ziyang Guo, Lina Wang, Shuang Hu, Yonggang Wang and Yongyao Xia**

Department of Chemistry and Shanghai Key Laboratory of Molecular Catalysis and Innovative Materials, Institute of New Energy, Collaborative Innovation Center of Chemistry for Energy Materials Fudan University, Shanghai 200433, China.

1. Figure S1. SEM images with different magnitudes of pristine VGCF.
2. Figure S2. TEM images with different magnitudes of pristine VGCF.
3. Figure S3. SEM images of leaf-like GO.
4. Figure S4 Characterizations: a) FT-IR spectrum b) Raman spectrum c) XRD pattern d) BET measurement.
5. Figure S5.Thermo-gravimetric (TG) analysis of leaf-like GO/S composites and GO/S composites.
6. Figure S6. Cycling performance of leaf-like GO/S composites with a sulfur content of 60 wt% at the rate of 0.2C (a) and 2C (b).
7. Figure S7 and S8. self-discharge investigation.
8. Figure S9. Comparison of cycling performances of conventional GO/S (45 wt%) and leaf-like GO/S (60 wt%) composite electrodes at the rate of 1C .
9. Figure S10. Voltage profile of the leaf-like GO/S composite cathode with different S-content in the composite.
10. Figure S11. Cycling performance at 0.5C of leaf-like GO/S composite cathodes with different S-content in the composites.
11. Figure S12. Voltage profile of the battery with different sulfur loading at rate of 0.5C.
12. Table S1 . "S-Loading vs. electrochemical performance" in different reports.

---

* Corresponding author. Tel & Fax: 0086-21-51630318
E-mail address: *ygwang@fudan.edu.cn*; *yyxia@fudna.edu.cn*

Figure S1

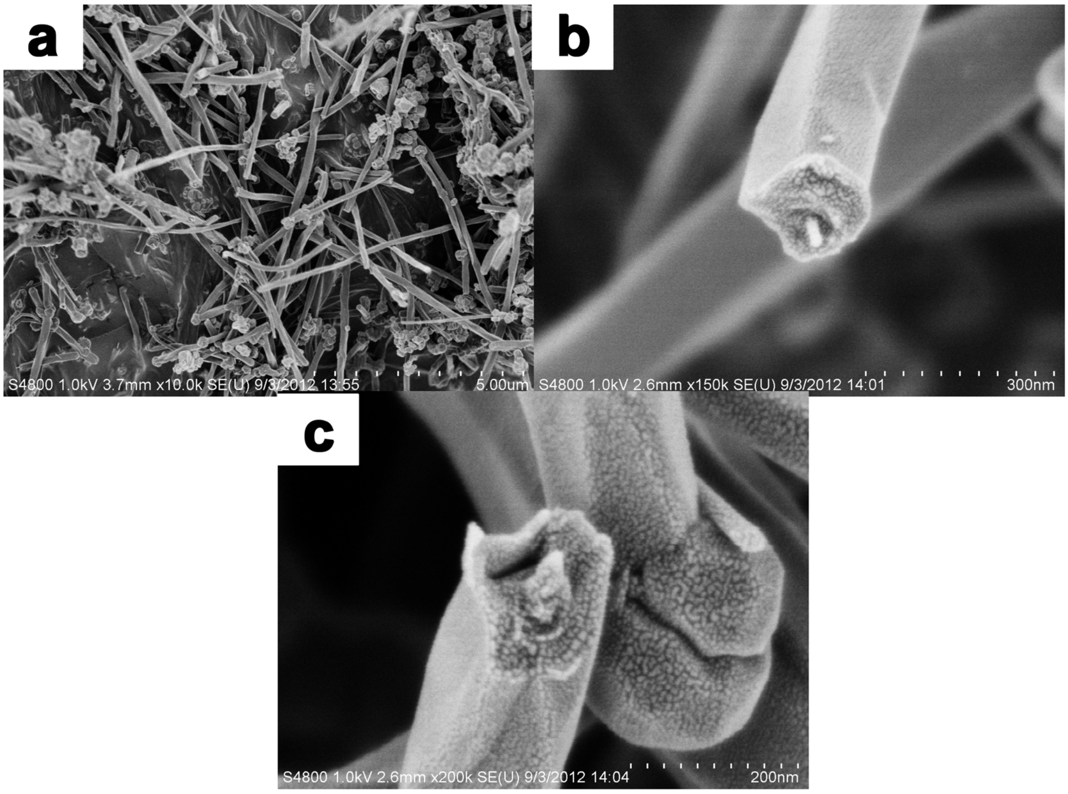

Figure S1. SEM images with different magnitudes of pristine VGCF. It can be detected that the diameter and length of the VGCF are ~150 nm and several microns (**Figure S1a**), respectively. Furthermore, it seems that the VGCF consists of spiral multi-layered graphene (**Figure S1b** and **S1c**), which will be further confirmed by TEM investigation (**Figure S2**). The amorphous particle-like materials, which can be seen in the **Figure S1a**, should be attributed to that the tops of VGCFs are anomalous and aggregated. Herein, it should be noted that the protuberance-like microstructure on the surface of VGCF arises from the gold sputtering for high resolution SEM observation.

Figure S2

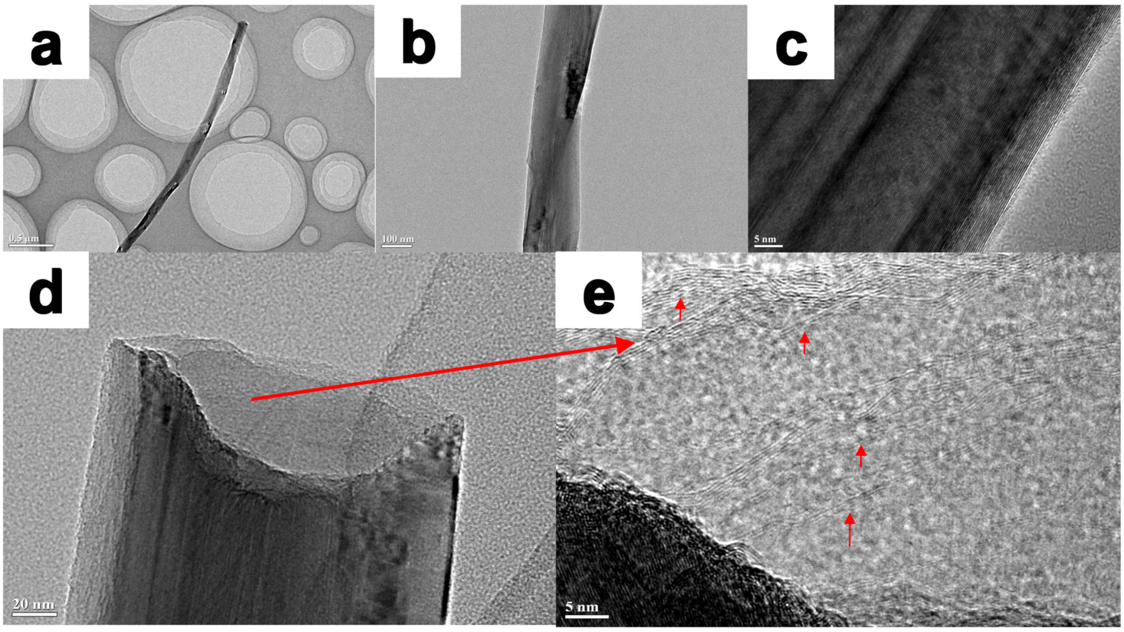

Figure S2.TEM images with different magnitudes of pristine VGCF. As shown in **Figure S2a** and **S2b**, the VGCF is typically around 120 nm, and can be up to several μm in length. According to TEM images of the top of VGCF (**Figure S2d** and **S2e)**, the structure of VGCF can be described as follow: multilayered graphite surrounds and wraps an axis of CNT to form a graphite rod.

Figure S3

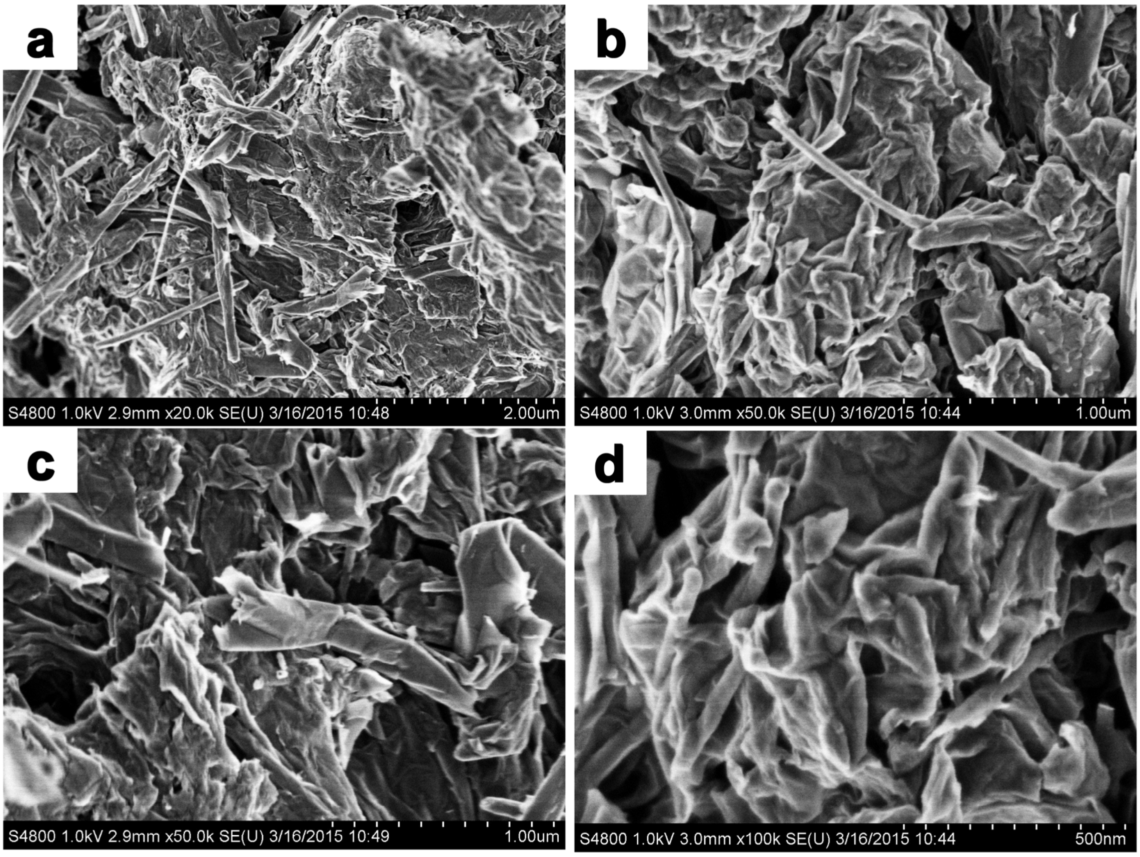

Figure S3. SEM images of leaf-like GO. As shown in **Figure S3** the spiral structure of VGCF has been peeled off to form leaf-like GO.

Figure S4

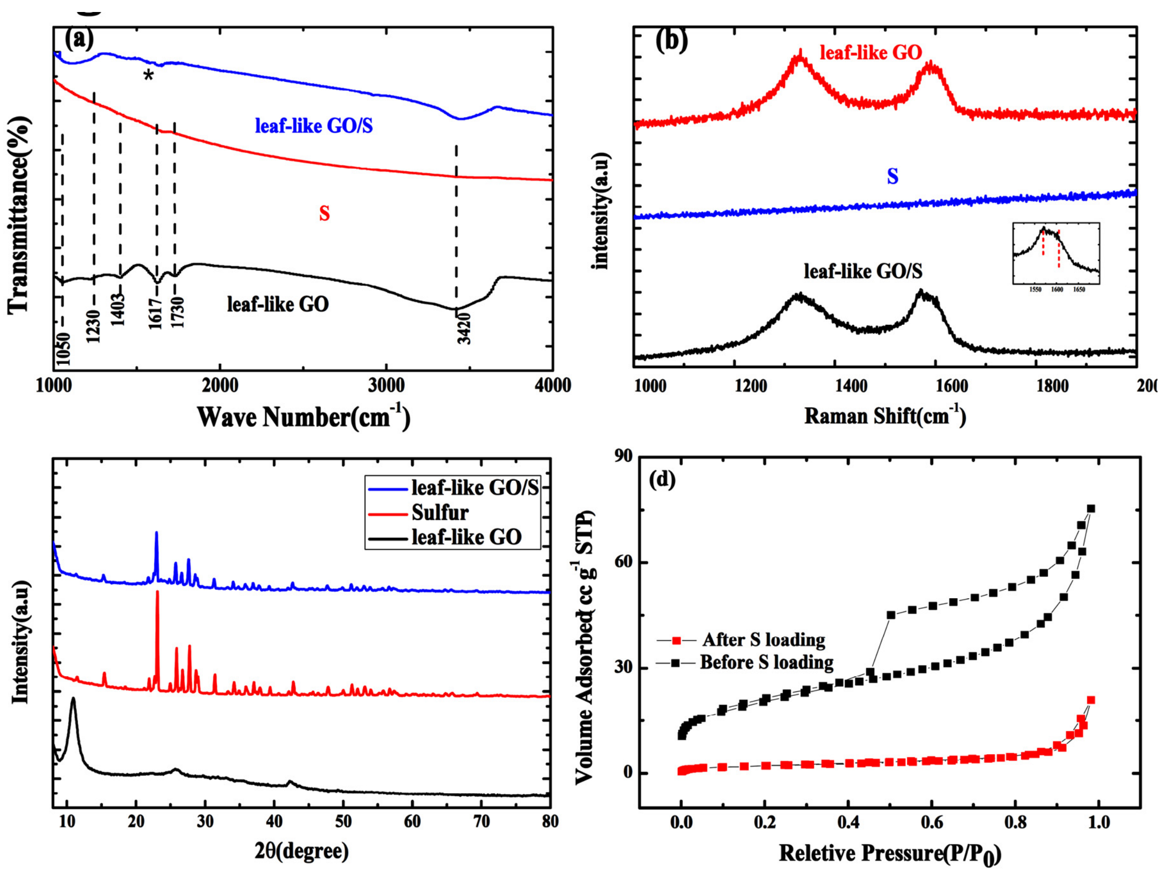

Figure S4. FT-IR Spectrum, Raman Spectrum, XRD pattern and the BET measurement. a) FTIR spectrum of leaf-like GO, S, leaf-like GO/S. b) Raman spectrum of leaf like GO, S, leaf-like GO/S. c) XRD pattern of S, leaf-like GO and leaf-like GO/S. d) BET measurement of leaf-like GO before(black) and after S loading(red).

To further Fourier Transform Infrared Spectroscopy (FT-IR), the Raman spectroscopy, X-ray diffraction(XRD) and Brunauer−Emmett−Teller (BET) analysis measurement were carried out to confirm the structure of leaf-like GO and leaf-like GO/S composites,. The Raman spectrum and FT-IR investigations of sulfur, leaf-like GO and leaf-like GO/S composites were recorded on a LabRAM HRRaman spectrometer using laser excitation at 514.5 nm and FT-IR (NICOLET 6700), respectively. The FTIR was measured in transmission geometry with KBr powder pellet. **Figure S4a** and **Figure S4b** exhibit the Fourier Transform

Infrared Spectroscopy (FT-IR) and the Raman spectroscopy characterization of leaf-like GO, bare sulfur and leaf-like GO/S composites. It can be identified in leaf-like GO by FT-IR spectrum: O−H stretching vibration (3420 cm^{-1}) associated with adsorbed water on the surface of GO, C=O stretching vibration (1730 cm^{-1}), C=C from unoxidized sp^2 CC bonds (1617 cm^{-1}), C−O−C (1250 cm^{-1}) from epoxy group vibration, C–O (1050 cm^{-1}) corresponding to the carbonyl vibration and carboxyl-associated O–H (1403 cm^{-1}).[RS1-RS3] The spectrum from bare S shows a rather smooth curve. However, in the spectrum of the leaf-like GO/S composites, some of these oxygen functional groups derived from the intensive oxidation disappears, indicating that GO has been partially reduced. Raman spectrum was also carried out on leaf-like GO, bare sulfur particles and leaf-like GO/S. Raman spectra of leaf-like GO shows a peak at 1350 cm^{-1}(tangential disorder-induced D-band) and a broad peak from 1540 cm^{-1} to 1650 cm^{-1} (G-band), which may be two overlapped peaks assigned to the coplanar vibration of sp^2-bonded carbon atoms of CNT midribs and GO respectively. The two overlapped peaks in the leaf-like GO are further confirmed by the Raman spectrum of leaf-like GO/S composite, which displays two overlapped peak at 1575 cm^{-1} and 1590cm^{-1} (see inset of **Figure S4b**). In leaf-like GO/S, a peak at 1350cm^{-1} (D-band) is assigned to the defects and disorder, while the two overlapped peaks at ~1575 and ~1590 cm^{-1} (G-band) are related to the coplanar vibration of sp^2-bonded carbon atoms of CNT midrib and GO/S.[RS4] It should be noted that the intensity of the peak at 1590 cm^{-1} in leaf-like GO/S becomes much weaker, indicating that some of the GO surface is coated with sulfur. In addition, the intensity ratio of D-band and G-band (I_D/I_G) of the leaf-like GO is approximately ~1.22, while in the leaf-like GO/S composites the intensity ratio of D-band and G-band (I_D/I_G) reduces to ~1.02. The result indicates that the carbon matrix in leaf-like GO/S composites is partially graphite due to the heat-treatment at 155℃, which would facilitate the electron transfer from/to the poorly conductive S. [RS4]

To further confirm the deposition of sulfur on the leaf-like GO composites, Powder X-ray diffraction and N2 adsorption isotherms were also carried out. Powder X-ray diffraction (XRD) measurements were performed on a NanoSTAR small-angle X-ray scattering system (Bruker, Germany) with Cu KR radiation. The Nitrogen adsorption/desorption analysis was done at 77 K on a Quantachrome AsiQwin. Before measurement, the samples were degased at 150℃ for leaf-like GO and room temperature for leaf-like GO/S composites. The specific surface areas were determined by the Brunauer−Emmett−Teller (BET) theory from the adsorption branch of the isotherm. **Figure S4c** gives the XRD pattern of S, leaf-like GO (inset in **Figure S4c**) and leaf-like GO/S composites. As shown in the **Figure S4c**, the leaf-like GO exhibits three peaks at ~10, ~25 and ~43 degree, which can be assigned to the (0,0,1) lattice plane of GO, (0,0,2) and (1,0,0) lattice plane of CNT respectively. The leaf-like GO/S composites exhibit a similar XRD pattern to S, indicating that the sulfur is deposited on the surface of leaf-like GO composites. As further comfirmed by the XRD pattern of leaf-like GO/S composites, the sulfur particles in the leaf-like GO/S composites are highly crystallized with Fddd orthorhombic structure. Notably, the sharp peak corresponding to the (0,0,1) lattice plane of GO at ~10 degree disappeared in the leaf-like GO/S composites, indicating that GO is partially reduced[RS5] owing to the heat treatment in argon at 155℃. In order to further characterize the pore structure of leaf-like GO and leaf-like GO/S composites, Brunauer－Emmett－Teller method was also carried out. **Figure S4d** shows the N_2 adsorption isotherms of both leaf-like GO and leaf-like GO/S composites. As shown in **Figure S4d**, the N_2 adsorption isotherms of the leaf-like GO (black curve) exhibits a typical-IV curve with a specific area 72.85 m^2 g^{-1}, suggesting the mesoporous structure with narrow pore size distribution. After loading sulfur on the leaf-like GO, the N_2 adsorption isotherms (red curve) became a typical type II curve. The result indicates that the pore on the surface of leaf-like GO has been filled with sulfur. Moreover, after S loading on the surface, the specific surface

area sharply reduces from 72.85 m^2 g^{-1} to 7.52 m^2 g^{-1}, the sharp decrease in the specific surface area indicate that the pore on the surface of leaf-like GO is filled with sulfur.

Figure S5

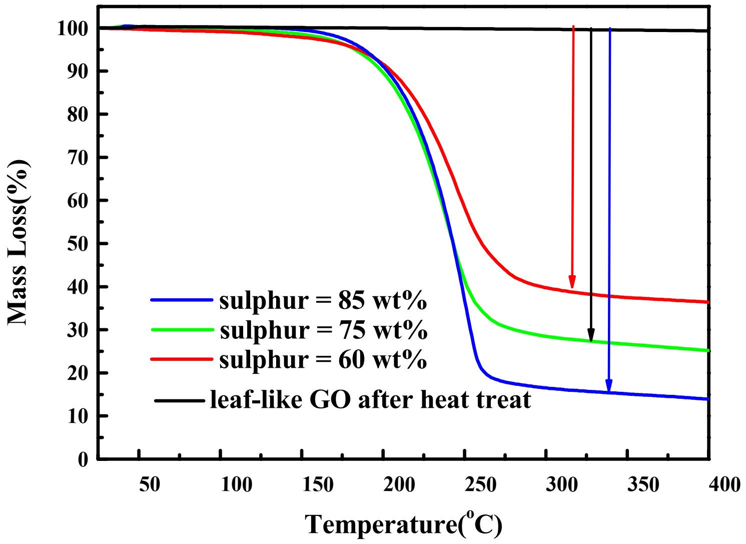

Figure S5. Thermo-gravimetric (TG) analysis of a) leaf-like GO/S composites with different sulfur contents. Thermo-gravimetric (TG) analysis was carried out on a Perkin–Elmer TGA 7 thermal analyzer with a heating rate of 5℃ min^{-1} in a 20 ml m^{-1} N_2 flow. The sulfur contents in the leaf-like GO/S composites are 60wt %, 75wt % and 85 wt% respectively. It should be noted that the weight loss due to the loss of functional groups on "the GO after heat treatment at 155℃" can be neglected below the temperature of 400℃, which is finely consistent with Zhang et al. previous reports [RS6]. Therefore, we are able to calculate sulfur content in leaf-like GO/S composites using TG analysis.

Figure S6

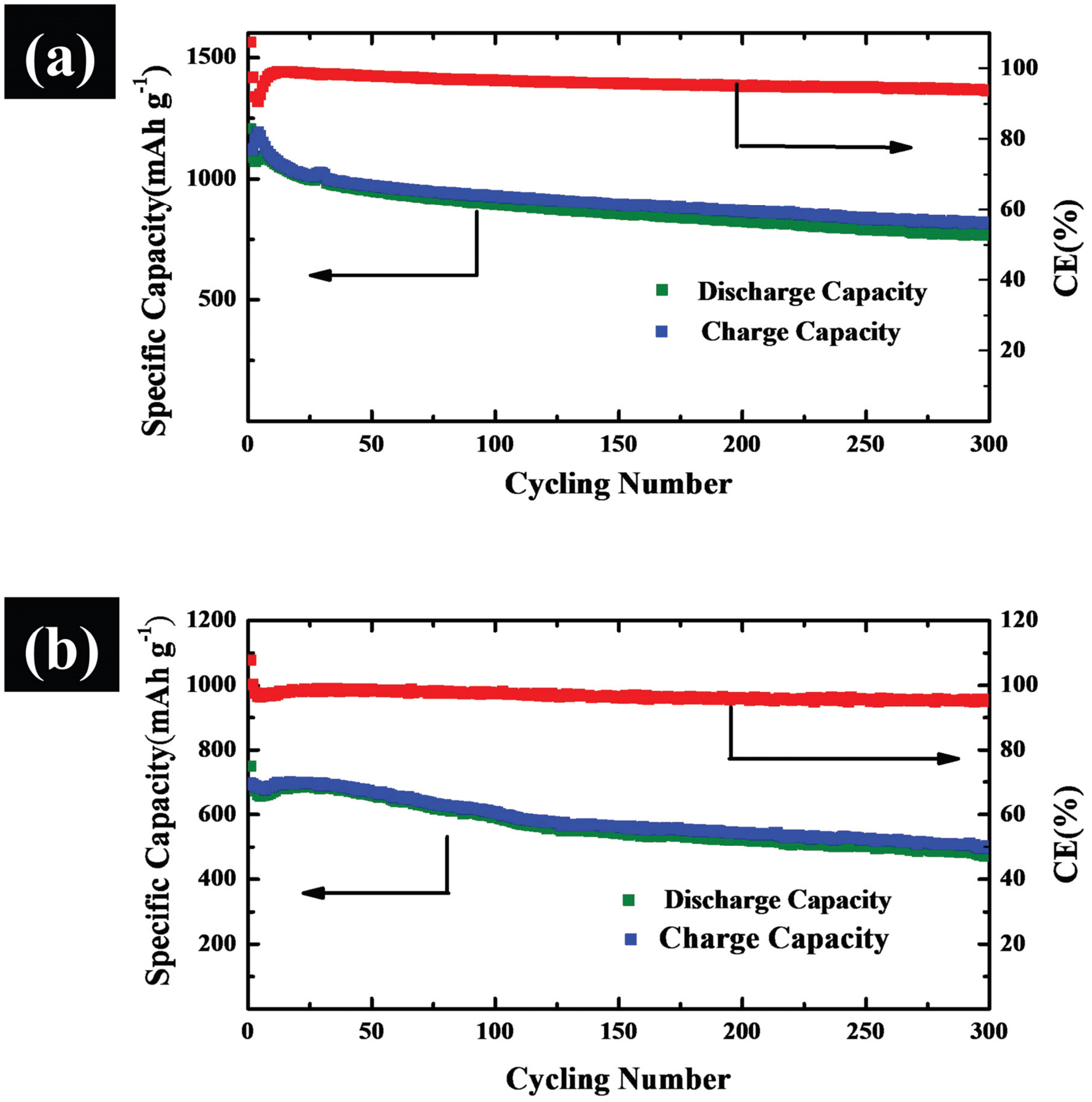

Figure S6. Cycling performance of leaf-like GO/S composites with a sulfur content of 60 wt% at the rates of a) 0.2C and b) 2C.

Figure S7

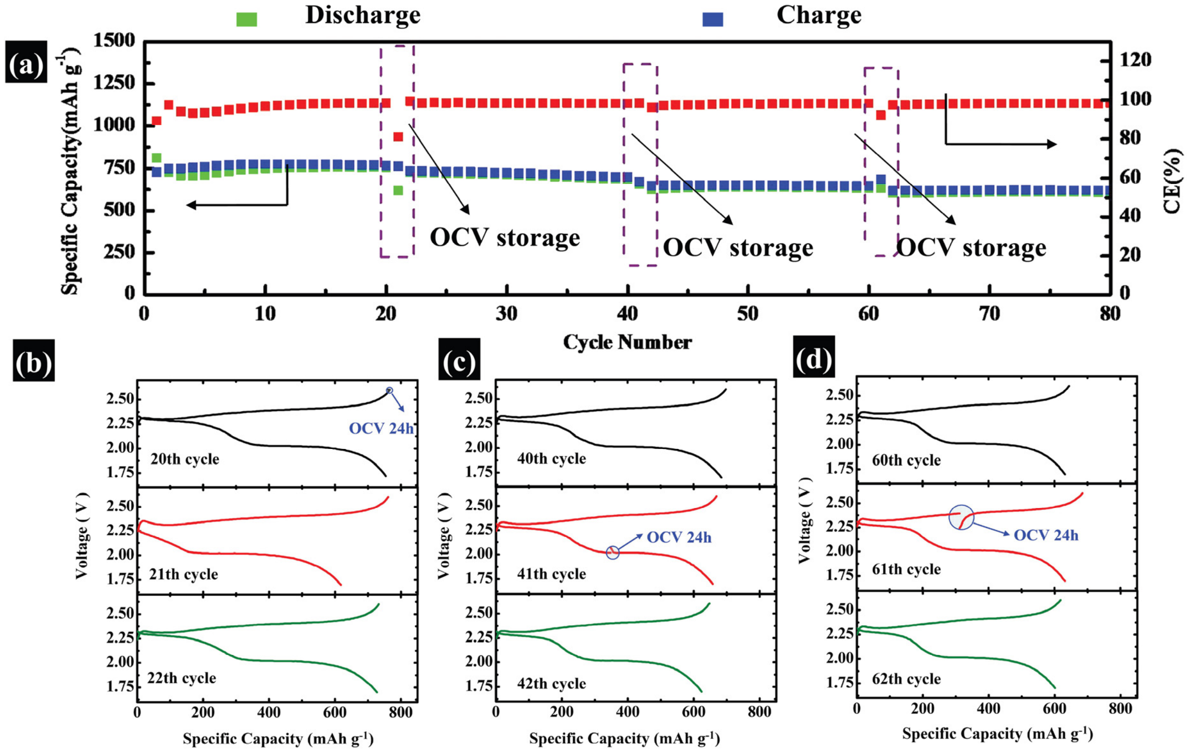

Figure S7. Self-discharge test of leaf-like GO/S cathode. a) Cycling performance with self-discharge tests at different states. b) Voltage profiles at full charge state for 24 hours at 20th cycle. c) Voltage profiles at half discharge state for 24 hours at 41th cycle. d) Voltage profiles of the battery rest at half charge states for 24 hours at 61th cycle.

To investigate the self-discharge of Li–S battery with leaf-like GO/S composites, we cycled a battery using leaf-like GO/S composites(S-content in the composites =60 wt%) as cathode at the rate of 1C for 19 cycles, and then the battery was rest for 24 hours at full charge state of 20th cycle (**Figure S7b**). The open circuit voltage during the rest was continuously measured by Land Cycler (see **Figure S8**). After that, the battery continued for discharge/charge test for 20 cycles (i.e. 21th -40th cycle). Then, the battery was rest for 24 hours at half discharge state

of 41th cycle (**Figure S7c**). Subsequently, the battery went on cycling for 20 times (i.e. 41th - 60th cycle) and then was rest for another 24 hours at half charge state of 61th cycle (**Figure S7d**). Finally, the battery was cycled for another 20 cycles (i.e. 61th -80th cycle).

As shown in **Figure S7b**, after rest for 24 hours at full charge state, the upper discharge plateau at 2.35V partly disappears, indicating the slow dissolving of sulfur and polysulfides in the electrolyte. The discharge capacity after rest for 24h dropped from 754mAh g^{-1} to 617 mAh g^{-1}. However, the discharge capacity almost recovered to 726 mAh g^{-1} after recharge, suggesting a good confinement of sulfur and polysulfides in cathode region. Then, we investigated the self-discharge at half discharge state after cycle for sequent 20 times. Notably, after rest for 24 hours at half discharge, the battery showed no capacity fading at 41th cycle (see **Figure S7c**). Finally, we investigated the self-discharge at half charge state after cycling the battery for another 20 cycles (i.e. 41st~ 61st cycle). After rest for 24 hours at half charge state, the battery continued to cycle without obvious capacity decay (**Figure S7d**).

Figure S8

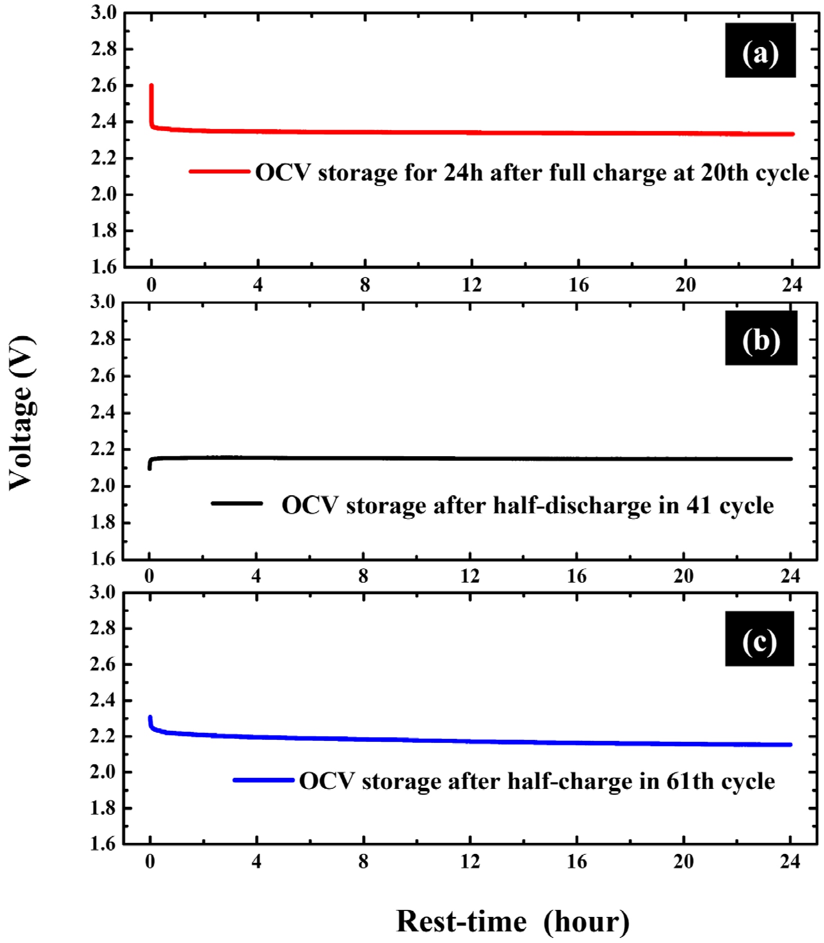

Figure S8. OCV for Self-discharge: a) OCV storage for 24h after full charge at 20th cycles. b) OCV storage for 24h after half-discharge at 41th cycle. c) OCV storage for 24h at half-charge at 61th cycle. During the rest time, the OCV shows no obvious change, indicating the limited self-discharge with leaf-like GO/S.

Figure S9

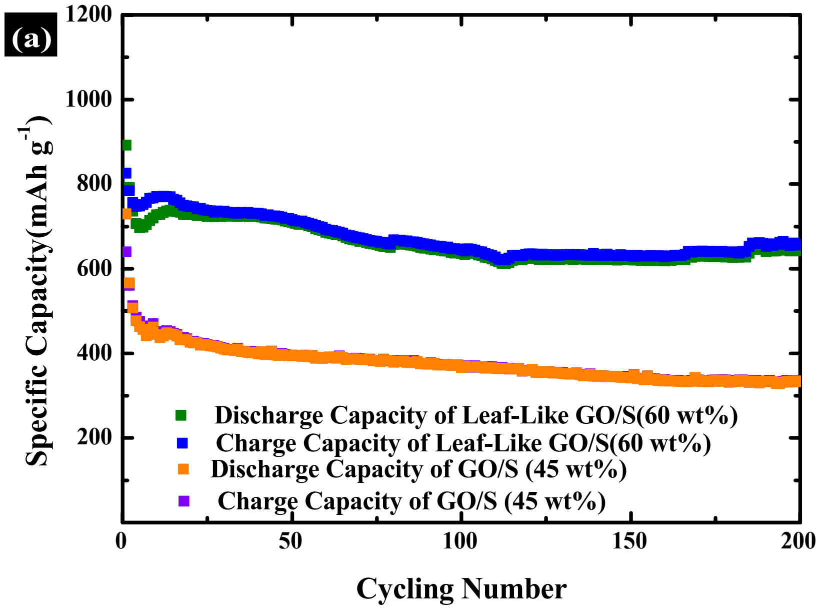

Figure S9. Comparison of cycling performances of conventional GO/S (45 wt%) and leaf-like GO/S (60 wt%) composite electrodes at the rate of 1C . For comparison, GO are also synthesized from graphite by a conventional Hummer's method. The conventional GO/S composites were also prepared by the same procedures. The sulfur content in the GO/S composites is 45 wt%. As shown in **Figure S9,** leaf-like GO/S (60wt%) and conventional GO/S composites show the similiar cycling performance, which should be attributed to the similar 2D structure of both GO and leaf-like GO to physically suppress the polysulfide shuttling effect and the identical functional groups on the surface of leaf-like GO/S composites and GO/S composites to bind the sulfur particles and its discharge products. However, much lower specific capacity of GO/S composites can be observed at the same rate even with lower sulfur content of 45 wt% in the composites. The low capacity of conventional GO/S composites mainly arises from the poor conductivity of conventional GO. Consequently,

the poor conductivity of conventional GO will lower the utilization of S in GO/S composites and deteriorate the rate performance of Li-S battery.

Figure S10

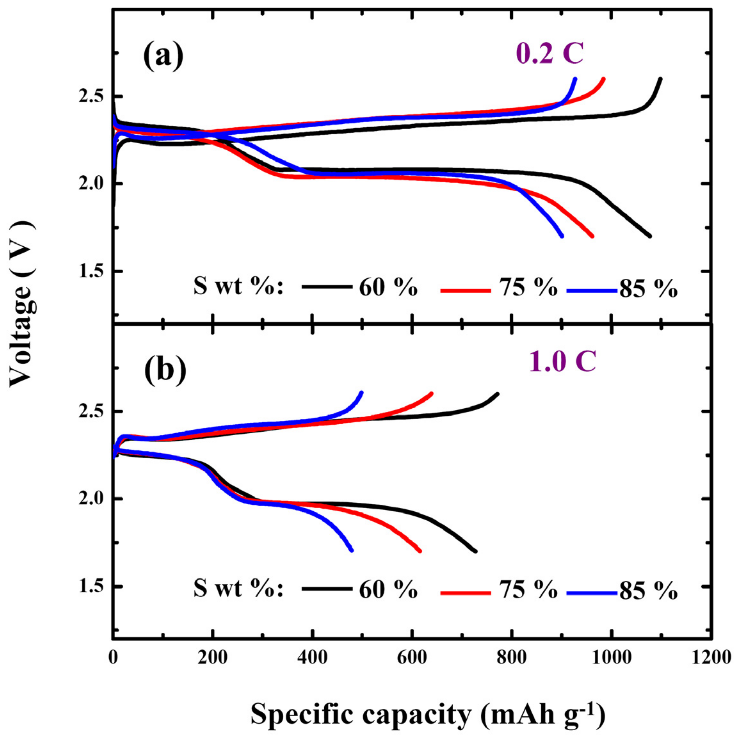

Figure S10. Voltage profile of the leaf-like GO/S composite cathodes with different S-content in the composites. a) Voltage profiles at the rate of 0.2C. b) Voltage profiles at the rate of 1C.

Figure S11

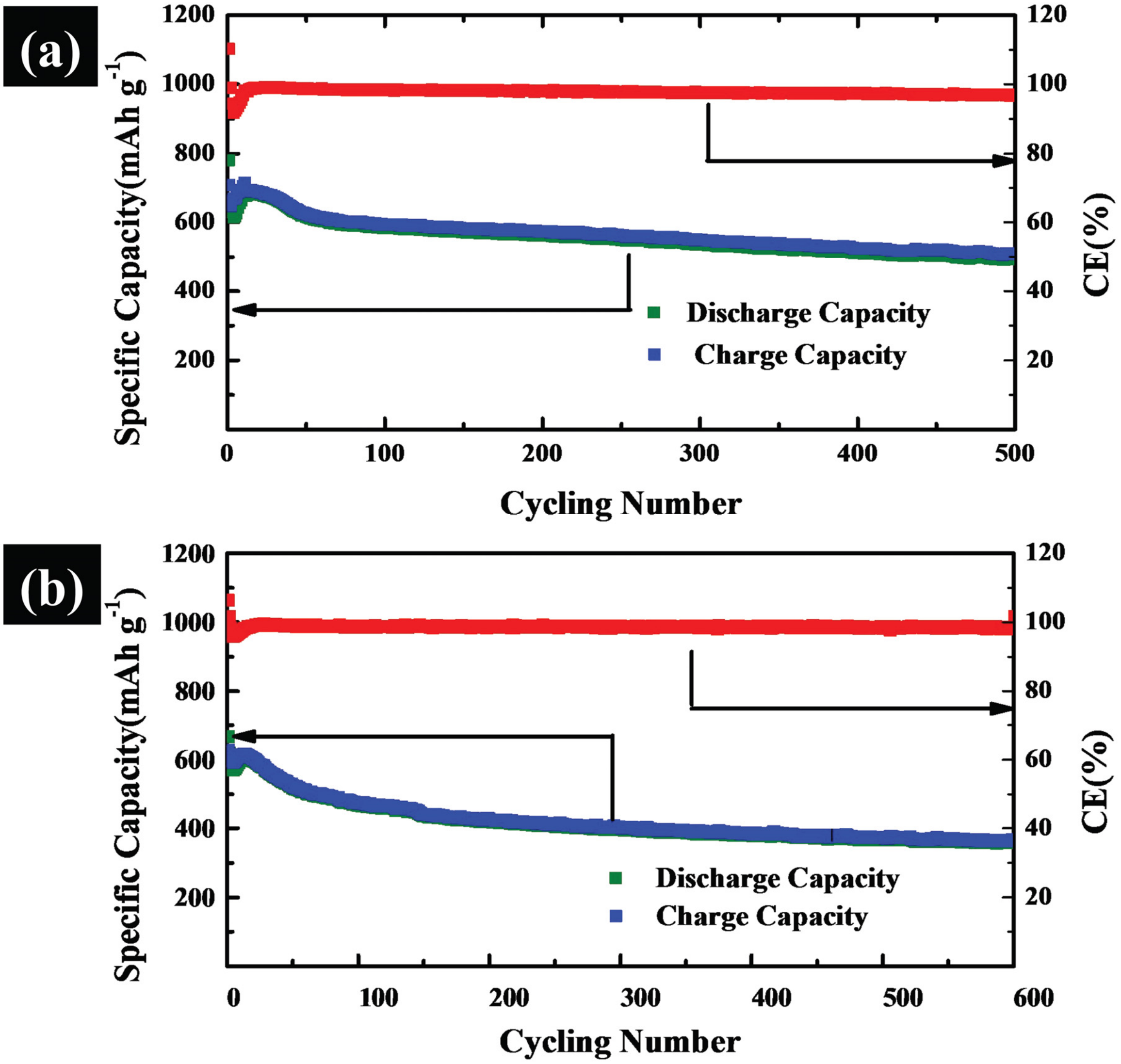

Figure S11. Cycling performance at 0.5C of leaf-like GO/S composite cathodes with different S-content in the composites. a) S-content of 75 wt%. b) S-content of 85 wt%. As shown in **Figure S11**, Both of the composites show long cycling performances over 500 cycles.

Figure S12

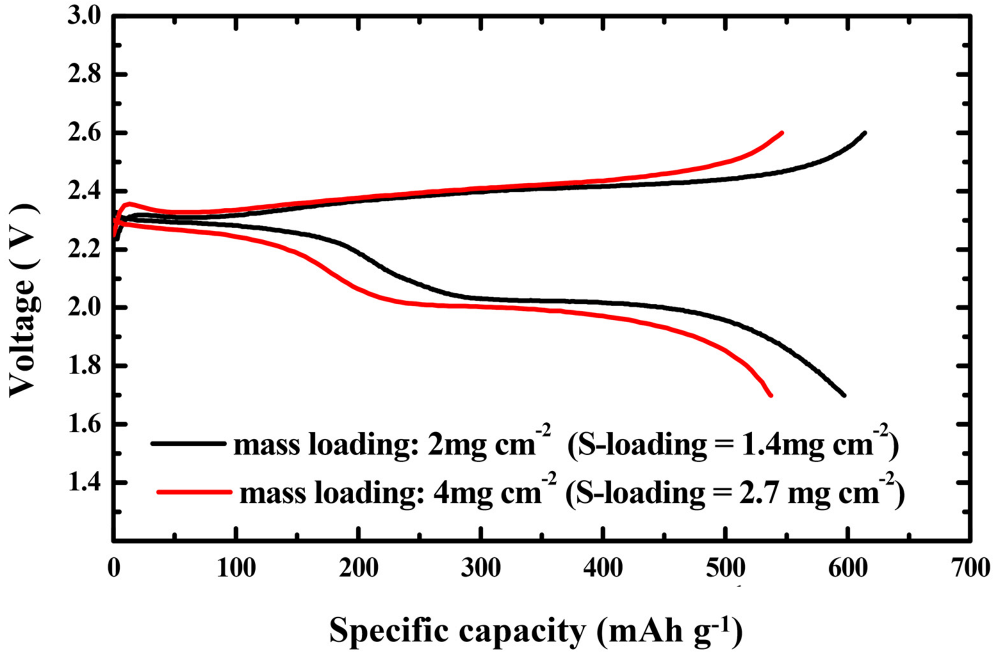

Figure S12. Voltage profile of the battery with different sulfur loading at rate of 0.5C.

Figure S12 displays the voltage profile of leaf-like GO/S composites (85 wt%) with total mass loading of 2mg cm^{-2} and 4mg cm^{-2} at both tenth cycles. Even with high sulfur content of 85 wt% and high sulfur loading of 2.7mg cm^{-2}, a capacity of 546 mAh g^{-1} is still achieved at the rate of 0.5C.

Table S1. "S-Loading vs. electrochemical performance" in different reports.

S loading [mg/cm^2]	Average Working Voltage[V]	Initial Areal Capacity [mAh/cm^2]	Cycle life [cycles]	Ref.
**2.7**	**~2.0**	**3.0**	**600**	**Present**
1.0	~1.7	0.8	200	13
1.1	~1.8	1.3	100	16
4.0	~2.0	3.3	100	18
**0.8**	**~2.0**	**1.2**	**500**	**20**
1.2	~2.0	1.6	100	21
**1.0**	**~1.3**	**0.9**	**500**	**23**
1.1	~2.0	1.7	100	26
0.8	~2.0	1.1	400	28
2.0	~2.0	2.7	100	29
1.1	~2.0	1.5	100	30
1.0	~2.0	2.2	80	36
0.43	~2.0	1.0	200	39
0.65	~2.0	1.1	100	40
**0.8**	**~2.0**	**1.2**	**1500**	**43**
**0.6**	**~2.0**	**0.5**	**1500**	**45**
**0.4**	**~2.0**	**0.6**	**1000**	**51**

As shown in **Table S1**, most excellent cycling performances over 500 cycles are achieved with very low sulfur loading **(⩽ 1mg/cm^2** on the electrode) with the special areal capacity lower than **1.5 mAh/cm^2**. Obviously, low sulfur loading on the electrode will facilitate the electrochemical performance of Li-S battery because of the low absolute current for test [i.e. (mA/g) × (g/cm2)]. However, for the practical application of Li-S batteries, to achieve the same specific areal capacity (2-3 mAh/cm^2) as Li-ion batteries do, reasonable sulfur loading on the electrode is essential [see review article: *J. Power Sources*, **2013**, 231, 153-162]. In our composites, even with high sulfur loading of 2.7 mg cm^{-2} on the electrode and specific areal capacity of 3.0 mAh/cm^2, the composites still show long cycling performance over 600 cycles at current rate of 0.5C, which is much better than previous reports. Herein, it should be

noted a lot of other previous reports does not mention the S mass loading in the experimental description, and thus are not included in **Table S1.**

References for supporting information:

Ref. S1 Y. Si, E.T. Samulski, *Nano Lett.* **2008**, 8, 1679 -1682.

Ref. S2 J.I. Paredes, S. Villar-Rodil, A. Martinez-Alonso, J. M. D Tascon, *Langmuir* **2008**, 24, 10560-10564.

Ref. S3 S.F. Peng, H.M. Cheng, *Carbon* **2012**, 50, 3210-3228.

Ref. S4 R.J. Chen,T. Zhao, J. Lu, F. Wu, L. Li, J.Z. Chen, G.Q. Tan, Y.S. Ye, K. Amine, *Nano Lett.* **2013**, 13, 4642−4649.

Ref. S5 Z.Y. Wang, Y.F. Dong, H.J. Li, Z.B. Zhao, H.B. Wu, C. Hao, S.H. Liu, J.S. Qiu, X.W. Lou, *Nat. Commun.* **2014**, 5**,** 5002.

Ref. S6 Ji L.W. L.W. Ji, M.M. Rao, H.M. Zheng, L. Zhang, Y.C. Li, W.H. Duan, J.H. Guo, E. J. Cairns, Y.G. Zhang, *J. Am. Chem. Soc.* **2011,** 133, 18522−18525
